# Comparison of ICU Mortality Rates Among Critically Ill COVID-19 Pneumonia Patients Across the First and Second Waves: A Single-Center Retrospective Analysis

**DOI:** 10.7759/cureus.77587

**Published:** 2025-01-17

**Authors:** Tejas VM, Veena RM, Mohan ME

**Affiliations:** 1 Internal Medicine, BGS Global Institute of Medical Sciences, Bangalore, IND; 2 Pharmacology, BGS Global Institute of Medical Sciences, Bangalore, IND

**Keywords:** ards, covid, covid-19, critical, death rates, first wave, icu, mortality rates, pandemic, second wave

## Abstract

Introduction: The experience gained from managing the first wave of the pandemic, combined with the abundance of scientific evidence, enabled physicians to identify risk factors for mortality outcomes and evaluate the effectiveness of respiratory support measures, vaccines, and drugs. This knowledge empowered them to confront the subsequent waves with greater expertise. Despite these advancements, the mortality rate among critical COVID-19 patients remained alarmingly high worldwide during these waves. It is uncertain whether advancements in critical care during the pandemic have improved outcomes for critically ill COVID-19 pneumonia patients admitted to the intensive care units (ICUs).

Methods: This was an observational, retrospective cohort study utilizing the collected data from COVID-19 patients between May to July 2020 and May to July 2021 at the Medical Records Department (MRD) section of BGS Global Institute of Medical Sciences Hospital, Bangalore, Karnataka.

Results: The mortality rate was higher among males in 2020 (66.1%) and 2021 (65.7%) compared to females in 2020 (33.9%) and 2021 (34.3%), with males accounting for nearly double the proportion of deaths in both waves. In 2020, the highest mortality was observed in the age group of 41-50 years (27.1%), while in 2021, it was noted in the age group of 51-60 years (24.3%). Altogether, patients in the age group of 41-60 years were the ones who were mostly affected in both waves. The average hospital stay increased from 3.8 days in the first wave to 7.4 days in the second. Diagnosis of COVID-19 pneumonia with comorbidities while being admitted into the ICUs accounted for 11.5% of cases in the first wave, rising to 68.6% in the second wave. Cause of death solely due to type 1 respiratory failure and acute respiratory distress syndrome was reported in 92.3% of cases in 2020 and 70.7% in 2021. In contrast, other non-respiratory causes contributed to 7.7% of deaths in 2020 and 29.3% in 2021.

Conclusion: This study provides insights into the ICU mortality trends in critically ill COVID-19 pneumonia patients during the first and second waves of the global pandemic. Despite advancements in disease knowledge, treatment protocols, and healthcare system management, no significant improvement in the mortality rates was found in our study. The mutations of the virus, which enhanced its transmissibility and immune escape characteristics, greatly contributed to our findings. Adoption of stringent social distancing, regular screening, early reporting of symptoms by the patient, well-established isolation, strict adherence to standard operating procedures (SOPs), and health advisory guidelines reduced ICU occupancy by patients diagnosed with COVID-19 pneumonia only, making room for complex cases to receive adequate timely care and treatment during the second wave. Limitations of the study include its single-center study design, missing data, and lack of post-vaccination data, all of which must be duly considered.

## Introduction

The hospitals and intensive care units (ICUs) were firmly hit by the first wave of COVID-19 resulting in the collapse of health systems due to uncertainty in the nature of the new disease and prognosis. The unpreparedness and resource shortage led to difficulty in confronting the pandemic in terms of insufficient ICU beds, ventilators, and personal protective equipment [1]. The first wave of the pandemic was characterized as a "single, massive wave," lacking an infection pattern typical of other viruses. The initial surge was partially contained through lockdown measures and social distancing strategies [2]. However, the relaxation of isolation measures and the spread of the virus led to a rise in infection rates, bringing the pandemic back with the onset of the subsequent waves. During subsequent waves, the scientific understanding of the disease was sharp, and consequently, healthcare systems engaged in delivering a better medical response, unlike the first wave.

The experience gained from managing the first wave of the pandemic, combined with the abundance of scientific evidence, enabled physicians to identify risk factors for mortality outcomes and evaluate the effectiveness of respiratory support measures, vaccines, and drugs. This knowledge empowered them to confront the subsequent waves with greater expertise. Despite these advancements, the mortality rate among critical COVID-19 patients remained alarmingly high worldwide during these waves. Limited data exist on the trends in mortality among critically ill COVID-19 patients in India throughout the duration of the pandemic.

There is a lack of studies analyzing ICU outcomes of COVID-19 patients across the entire first year of the pandemic to assess variations between different waves [2]. Therefore, we started a retrospective pilot study in our ICU, aiming to compare mortality trends between the first and second waves of the pandemic among critically ill COVID-19 pneumonia patients admitted to the ICU.

## Materials and methods

This was an observational, retrospective cohort study utilizing the collected data from COVID-19 patients between May to July 2020 and May to July 2021 at the Medical Records Department (MRD) section of BGS Global Institute of Medical Sciences Hospital, Bangalore, Karnataka. The above-mentioned time frames were considered as first and second waves, respectively, as the maximum incidence of COVID-19 cases was observed during this period in our healthcare setup. A comprehensive database was established through the consecutive enrolment of critically ill COVID-19 patients. Inclusion criteria comprised adults admitted to any participating ICU who met the diagnosis of COVID-19 pneumonia with acute respiratory failure. For this study, only patients with missing outcome data were excluded. Waiver of informed consent was granted as all collected data were de-identified by omitting patient names and medical record numbers.

Data collection

Patient demographic and clinical data were documented in a case report form. The recorded information included demographic details (age, gender), comorbidities, illness timeline (dates of symptom onset, diagnosis, hospital admission, and ICU admission), complications, and treatments administered [3]. These data were analyzed to assess outcomes. Data on drug protocols, prophylactic antithrombotic agents, and anticoagulants were also collected, although the treatment protocols for patients admitted in 2020 and 2021 followed those issued by the Indian Council of Medical Research (ICMR).

Ventilation

Respiratory support was administered in accordance with local standardized protocols, with the attending physician tailoring settings based on real-time monitoring of cardiorespiratory parameters [4].

Steroids

Methylprednisolone was administered at a dosage of 2 mg/kg/day to prevent the development of pulmonary fibrosis in patients with a partial pressure of oxygen (PaO2)/fraction of inspired oxygen (FiO2) ratio of less than 150 for at least seven days while on mechanical ventilation [4].

During the second wave, steroid therapy typically involved dexamethasone at 6 mg intravenously (IV) per day for seven to 10 days, following the ICMR protocol. In cases where clinical worsening or a persistent hyperinflammatory state indicated inadequate response to initial glucocorticoid therapy, the treatment was shifted to methylprednisolone at 0.5 mg/kg IV every six hours [4].

Anticoagulants

Enoxaparin was administered subcutaneously at a dose of 40 mg every 12 hours as a prophylactic measure, with adjustments made based on the patient’s body weight. Unfractionated heparin was considered as an alternative in cases where a pulmonary embolism was confirmed via computed tomography (CT) scan or when there was a strong clinical suspicion [4].

Respiratory failure

In this study, respiratory failure was defined as arterial oxygen tension (PaO₂) < 60 mmHg (hypoxemic respiratory failure) or arterial carbon dioxide tension (PaCO₂) > 50 mmHg with pH < 7.35 (hypercapnic respiratory failure) on arterial blood gas analysis, consistent with the American Thoracic Society and European Respiratory Society guidelines.

Non-respiratory failure

In this study, non-respiratory failure causes of death were defined as all cases where the cause of death did not fulfill the criteria for respiratory failure as outlined in the study protocol. Such cases encountered in the study include decompensated chronic liver disease, organophosphate compounds, septic shock, cardiopulmonary arrest, subdural hemorrhage, acute kidney injury, multi-organ dysfunction syndrome, uremic encephalopathy, hypoxic ischemia encephalopathy, and hepatic encephalopathy.

Outcomes

Primary Outcomes

To compare mortality outcomes in COVID-19 pneumonia patients admitted to the ICU during the first and second waves.

Secondary Outcomes

To compare the length of hospital stay and the causes of death between the first and second waves.

Statistical analysis

**Figure 1:**
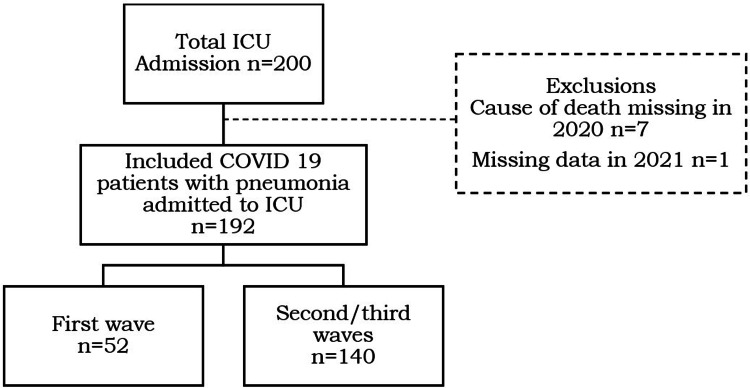
Flowchart of the study.

This study did not include a statistical sample size calculation. Instead, the sample size was determined by the total number of patients admitted to the participating ICUs throughout the study period. Descriptive statistics of the explanatory variables and the outcome variables were calculated by frequency, average, and proportions. Due to challenges in data collection during the pandemic, missing data were encountered, and the proportions of missing data are provided in the analysis (Figure 1).

## Results

During the first wave of COVID-19, from May 1, 2020, to July 31, 2020, a total of 59 participating patients with confirmed COVID-19 pneumonia who met the inclusion criteria were included in the study. Specific data on the cause of death were unavailable for May 2020. Missing data were thus excluded from the analysis. Hence, the analysis for the cause of death was based on 52 patients (n = 52), while all other parameters included data from 59 patients.

Similarly, during the second wave of COVID-19, from May 1, 2021, to July 31, 2021, a total of 141 patients participated in the study. One patient was excluded from the analysis due to missing data, resulting in a final sample size of 140 (n = 140) for all parameters in 2021.

Mortality rates by gender

**Figure 2:**
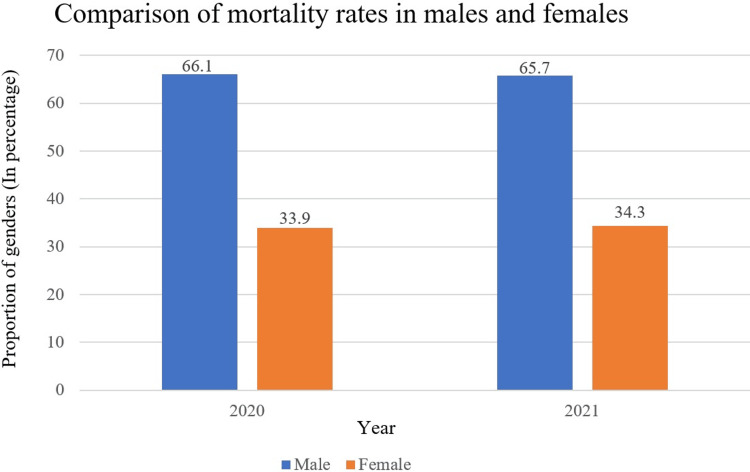
Mortality rates for different genders.

The mortality rates among males were higher, with 39 (66.1%) in the first wave and 92 (65.7%) in the second wave, compared to females, who had 20 (33.9%) in the first wave and 48 (34.3%) in the second wave, accounting for nearly double the number of male patients in both waves (Figure 2).

Mortality rates by age groups

**Figure 3:**
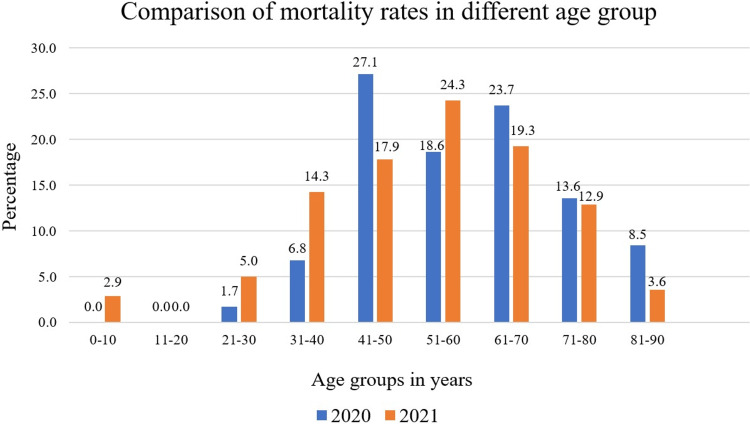
Mortality rates for different age groups.

In the first wave, the highest mortality rate was observed in the age group of 41-50 years, with 16 (27.1%) deaths. In the second wave, the highest mortality rate was noted in the age group of 51-60 years, with 34 (24.3%) deaths. Overall, mortality was highest in the age group of 41-70 years, irrespective of the COVID-19 wave (Figure 3).

The graph shows mortality rates for different age groups for two different waves. In the first wave, patients above the age group of 21 years were affected by COVID-19 with pneumonia whereas in the second wave, newborn babies were also affected. However, in both waves, no data were found for the age group of 11-20 years. So, in the graph, the age groups of 11-20 years were omitted (Figure 3).

Average duration of hospital stay

**Figure 4:**
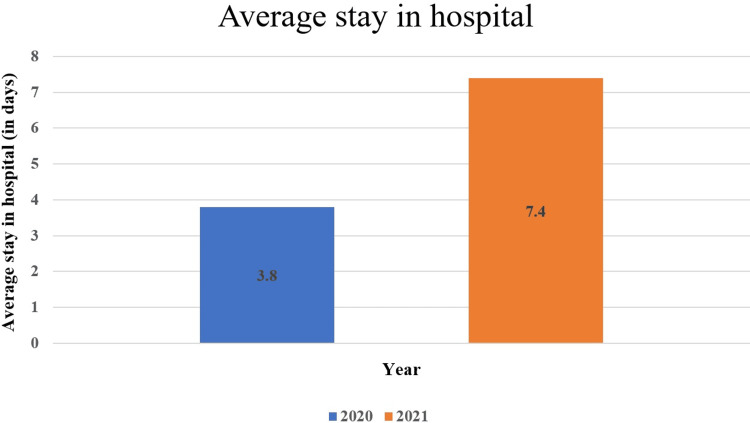
Average duration of hospital stay of COVID-19 patients with pneumonia.

The average duration of hospital stay was 3.8 days and 7.4 days in the first and second waves, respectively (Figure 4).

COVID-19 with and without comorbidities

In the first wave, among the 52 patients diagnosed with COVID-19 pneumonia, 46 (88.5%) had no underlying comorbidities, while six (11.5%) had some form of underlying comorbidities.

**Figure 5:**
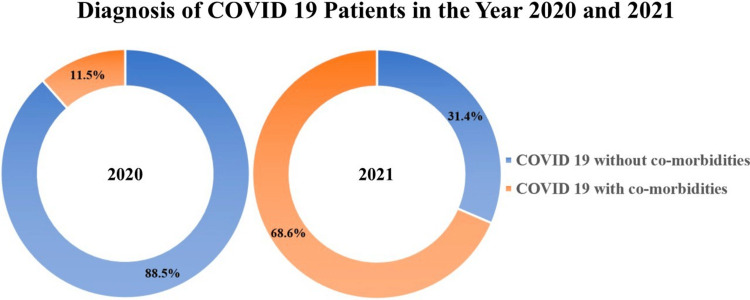
Diagnosis of patients admitted to the ICU with and without comorbidities in 2020 and 2021.

In the second wave, out of 140 patients diagnosed with COVID-19 pneumonia, 44 (31.4%) had no comorbidities, while the remaining 96 (68.6%) had comorbidities associated with their ICU admission (Figure 5).

Cause of death

**Figure 6:**
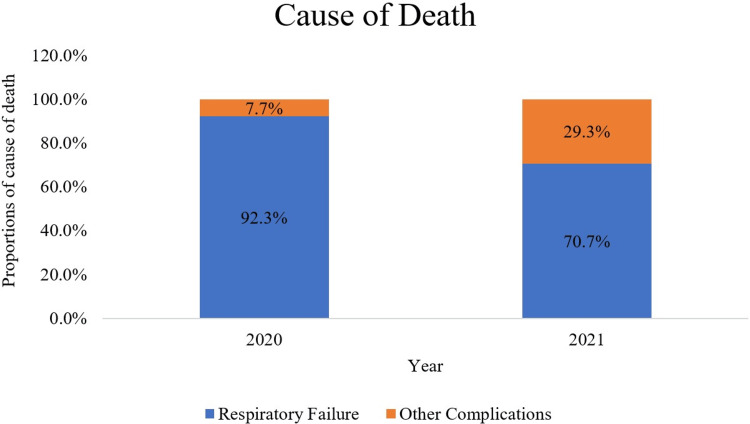
Proportion of causes of death in COVID-19 patients admitted to the ICU in the year 2020 and 2021.

The cause of death due solely to type 1 respiratory failure and acute respiratory distress syndrome was reported in 48 (92.3%) patients in 2020 and 99 (70.7%) patients in 2021. In contrast, four (7.7%) patients in 2020 and 41 (29.3%) patients in 2021 died due to other non-respiratory failure causes (Figure 6).

## Discussion

From this retrospective cohort study conducted in our setting involving critically ill COVID-19 pneumonia patients, a drastic rise in the admission of patients into the ICU was observed, the majority of them being males irrespective of the COVID-19 waves. The male mortality rate almost doubled that of the female in both waves.

Overall, in both waves, the age group of 41-70 years was affected more and showed a maximum mortality rate. Our findings differ from other studies that observed higher mortality outcomes among patients aged more than 70 years [2,5,6]. In the second wave, children below the age of 10 years were also affected, and among them, the majority were newborns (Figure 3).

Despite the improvement in healthcare standards, management, and treatment modalities realized after the experience gained by the physicians from the first COVID-19 wave, the average stay in the hospital of patients increased from 3.8 days in the first wave to 7.4 days in the second wave.

The cause of death remained to be due to respiratory failure predominantly irrespective of the waves. In contrast, our findings differ from those of previous studies, which reported a reduction in the median length of hospitalization during the second wave [5].

In our study, it was observed that patients diagnosed solely with COVID-19 pneumonia (88.46%) and without comorbidities (11.54%) were admitted to the ICU during the first wave. In contrast, during the second wave, there was an increase in ICU admissions of patients diagnosed with comorbidities (68.57%) along with COVID-19 pneumonia, accounting for a large proportion of ICU occupancy. From this, we can speculate that most of the COVID-19 cases were detected early and treated in the second wave effectively without allowing its progression to complicated states, which could have led to ICU admission.

Possible explanations for increased mortality rates in the second wave in our study may be due to mutations in the virus, which enhanced its transmissibility and immune escape characteristics, and large-scale migrations occurring during the waves, which challenged the lockdown and isolation measures [7]. On the part of healthcare systems, a lack of knowledge regarding the mutated variants of the virus causing the disease, shortage of oxygen, and the presumed inefficiency of the healthcare system in managing the caseload burden, straining the ICU capacity, may have been the reasons for the higher death toll during the second wave.

Stringent social distancing, regular screening, early reporting of symptoms by the patient, well-established isolation, strict adherence to standard operating procedures (SOPs), and health advisory guidelines may have majorly contributed to the control of disease from worsening despite the high infectivity of the virus during the second wave caused due to mutations in the virus strain, as studied by Tian et al. (2021). As a result, only the ones who were associated with other comorbidities ended up in the ICU with multisystem involvement than just COVID-19 pneumonia.

Strengths of the study

Interpreting changes in mortality rates throughout the global pandemic can be difficult due to differences in the timing of the COVID-19 waves and the wide range of underlying medical conditions present in patients. Data on mortality trends among critically ill COVID-19 patients in India throughout the pandemic are limited. There is a lack of studies analyzing ICU outcomes of COVID-19 patients across the entire first year of the pandemic to assess variations between different waves. With no large data available in an Indian healthcare setting, this study becomes important for considerations in such variable outcomes observed across the world marking its strength. Most available data focus on mortality trends over short study periods.

Limitations of the study

The study was carried out prior to widespread vaccination drives, and expedited immunization efforts may have significantly contributed to the reduction in mortality rates. More detailed data on the specific causes of death in COVID-19 are essential for a better interpretation of mortality rates. Missing data in clinical research can profoundly influence results, leading to potential biases and a decrease in the power of statistical analysis. In conclusion, although clinical experience, knowledge, and treatment strategies for managing this challenging infectious disease have advanced, it is essential to persist in the development of more effective treatments. Additionally, further research into the best supportive care practices is crucial to improving outcomes for critically ill COVID-19 patients.

## Conclusions

This study provides insights into the ICU mortality trends in critically ill COVID-19 pneumonia patients during the first and second waves of the global pandemic. Despite advancements in disease knowledge, treatment protocols, and healthcare system management, no significant improvement in the mortality rates was found in our study. The mutations of the virus, which enhanced its transmissibility and immune escape characteristics, greatly contributed to our findings. Adoption of stringent social distancing, regular screening, early reporting of symptoms by the patient, well-established isolation, strict adherence to SOPs, and health advisory guidelines reduced ICU occupancy by patients diagnosed with COVID-19 pneumonia only, making room for complex cases to receive adequate timely care and treatment during the second wave. Limitations of the study include its single-center study design, missing data, and lack of post-vaccination data, all of which must be duly considered.
